# Relevance of serum levels of the endoplasmic reticulum stress protein GRP78 (glucose-regulated protein 78 kDa) as biomarker in pulmonary diseases

**DOI:** 10.1007/s12192-023-01341-0

**Published:** 2023-04-05

**Authors:** Muntadher Al Zaidi, Vanessa Marggraf, Elena Repges, Georg Nickenig, Dirk Skowasch, Adem Aksoy, Carmen Pizarro

**Affiliations:** Department of Internal Medicine II, University Hospital Bonn, Venusberg-Campus 1, 53127 Bonn, Germany

**Keywords:** Endoplasmic reticulum stress, Chaperone, GRP78, Biomarker, Inflammation, Lung

## Abstract

Cellular stress and inflammation contribute to the initiation and progression of a variety of pulmonary diseases. Endoplasmic reticulum (ER) stress and its main regulator GRP78 (glucose-regulated protein 78 kDa) appear to be involved in the pathogenesis of pulmonary diseases, and GRP78 was found to be a biomarker in a wide range of inflammatory diseases. The aim of this study was to investigate the relevance of serum GRP78 in pulmonary disorders.

In this prospective cohort study, 78 consecutive patients with chronic obstructive pulmonary disease (COPD, *n* = 28), asthma (*n* = 38) or interstitial lung disease (ILD, *n* = 12) underwent measurement of serum GRP78 levels by ELISA.

The mean age of patients was 59.8 ± 12.4 years, 48.7% were female. Patients with elevated GRP78 levels (> median) offered a significantly better oxygenation status (capillary pO2: 75.3 ± 11.7 mmHg vs. 67.8 ± 15.9 mmHg, *p* = 0.02). Significant correlations were observed between GRP78, on the one hand, and haemoglobin, high-sensitivity C-reactive protein (hs-CRP) and eosinophil counts, on the other hand (haemoglobin: Pearson’s *r* = −0.25, hs-CRP: *r* = 0.30, eosinophils: *r* = 0.63).

Subsequently, we evaluated GRP78 measurements in function of severity stratifiers of the specific underlying pulmonary disease. ILD patients with a severe diffusion impairment (DL_CO_< 40% of predicted), exhibited a significant decrease in GRP78 levels (*p* = 0.01). In COPD and asthma, both characterized by obstructive ventilatory defects, a forced expiratory volume in one second (FEV_1_) <30% of predicted was accompanied by significantly lower GRP78 (*p* = 0.0075).

In both obstructive and restrictive pulmonary disorders, GRP78 protein concentrations were reduced with increasing disease severity. These data suggest a prevalent role of GRP78 in the presently studied pulmonary disorders.

## Introduction

The endoplasmic reticulum (ER) plays a crucial role in protein synthesis, correct folding and posttranslational modification of proteins (Luo & Lee, 2013). ER stress, resultant from a mismatch between ER protein folding capacity and load, leads to an accumulation of misfolded proteins in the ER (Chaudhari et al., 2014). Such an impairment in ER homeostasis exerts a major impact on cellular survival and function (Dastghaib et al., 2021). This, in turn, activates the unfolded protein response (UPR) signaling pathway in order to mitigate proteotoxic effects in stressed cells (Walter & Ron, 2011). However, in case of severe and prolonged ER stress, the UPR activates pathways that may elicit cell death through apoptosis (Wu & Kaufman, 2006).

The molecular chaperone glucose-regulated protein 78kDa (GRP78), a heat shock protein also known as binding immunoglobulin protein or heat shock protein A5, is the key mediator of the UPR (Lee, 2014). Its main function consists of restoring ER integrity via facilitating protein folding. In addition, an increasing number of recently published studies described secretion and extracellular functions of GRP78.

UPR dysregulation has been linked to numerous illnesses such as obesity, cancer and cardiovascular diseases (Dos Santos et al., 2023; Meyer & Doroudgar, 2020; Pan et al., 2022). In a study conducted by our group, we found reduced serum levels of GRP78 to predict mortality in patients with aortic valve stenosis undergoing transcatheter valve replacement (Aksoy et al., 2021). In another study, we provided evidence that elevated levels of GRP78 are linked to favourable clinical and hemodynamical parameters in patients with pulmonary arterial hypertension. Moreover, using *in vitro* models, we demonstrated a protective role of extracellular GRP78 in mediating pulmonary artery smooth muscle cell remodelling (Al Zaidi et al., 2022).

In chronic and debilitating respiratory disorders, the value of ER stress-UPR axis imbalances remain less well defined. Inhaled environmental stimulants, such as cigarette smoke and allergens, are known to induce ER stress and UPR dysregulation. Due to their exposition to a large number of airborne pathogens, human lungs are notably vulnerable to conditions of unresolved ER stress. A proteomics approach to biomarker in chronic obstructive pulmonary disease (COPD) identified several proteins, including GRP78, to be increased in COPD patients (Merali et al., 2014). They may reflect lung damage and airway remodelling and may potentially serve as COPD biomarkers. To assess cigarette smoke–induced lung injury, secretion of GRP78 was assayed in bronchoalveolar lavage fluid (Aksoy et al., 2017). Chronic smokers exhibited an enhanced GRP78 secretion as compared to non-smokers, supporting the notion of a GRP78 upregulation in the setting of lung oxidant stress. In idiopathic pulmonary fibrosis, emerging evidence implicates ER stress both as a cause and consequence of pulmonary inflammation and fibrosis (Dickens et al., 2019).

Given the continuous exposure of pulmonary cells to numerous environmental triggers, the aim of the present study was to investigate the potential of GRP78 as a biomarker in a cohort of patients comprising COPD, asthma and interstitial lung disease (ILD).

## Methods

### Study population

Between December of 2020 and April of 2021, a total of 78 consecutive patients aged ≥18 years who received treatment at the Department of Pneumology, University Hospital Bonn (Bonn, Germany), for COPD, asthma or ILD were enrolled in this prospective cohort trial. Patients were approached during their outpatient consultation, all patients presented stable disease without clinical exacerbation at the time of study inclusion. COPD diagnosis was spirometrically and clinically confirmed in line with the Global Initiative for Chronic Obstructive Lung Disease (GOLD) guidelines (Global Strategy for Prevention, 2023). Asthma diagnosis complied with the Global Initiative for Asthma (GINA) diagnostic criteria (*Global Initiative for Asthma: 2022 Main Report*, 2023). ILD diagnosis relied on consensus by multidisciplinary discussion, according to current guidelines (Raghu et al., 2022). All patients underwent blood sampling and pulmonary function testing. Each subject completed demographic and medical history questionnaires. The study was conducted according to the principles of the Declaration of Helsinki and was approved by the local ethics committee at the Medical Faculty of the University of Bonn. Written informed consent was obtained from all subjects prior to entry into the study.

### Blood specimens

Blood samples were collected during patients’ routine outpatient visit. Samples were kept on ice and centrifugated at 1500 × g for 15 min. Subsequently, serum was transferred into coded aliquots and stored at −80°C. Analysis of GRP78 levels (Enzo Life Sciences, Inc, Farmingdale, NY, USA) was conducted by commercially available enzyme-linked immunosorbent assay (ELISA) kits. 4-Parameters Logistic Regression (Graphpad Prism 9.0.0) was applied to analyse GRP concentrations.

In addition to GRP measurement, blood samples were acquired to assess full blood count and high-sensitivity C-reactive protein (hs-CRP) levels.

### Pulmonary function testing and capillary blood gas analysis

Pulmonary function testing was performed in conformity with the European Respiratory Society guidelines (Graham et al., 2019) and included spirometry, bodyplethysmography and determination of diffusion capacity for carbon monoxide (Bodyplethismograph Jaeger©, Alveo-Diffusionstest Jaeger©, Wuppertal, Germany). Static and dynamic lung volumes, such as forced expiratory volume in one second (FEV_1_), Tiffeneau-index (FEV_1_/VC) and diffusion capacity for carbon monoxide (DL_CO_) as assessed by the single-breath technique were analysed. Parameters were adjusted to standard population-derived predicted values.

By capillary blood gas analysis with a sample collected from the hyperaemic earlobe, partial pressure of oxygen (pO_2_) and partial pressure of carbon dioxide (pCO_2_) were determined.

### Statistical analysis

Continuous data are presented as mean ± standard deviation — if normally distributed — or as median and interquartile range (IQR, quartile 1/quartile 3) — if not normally distributed. Continuous variables were tested for having a normal distribution by use of the Kolmogorov–Smirnov test. Categorical data are given as absolute numbers and percentages. In the case of continuous parameters, Student’s *t*-test or Mann–Whitney *U-*test (if normality assumption was violated) was employed for comparison between two groups. Categorical variables were analysed by Fisher´s exact test. Correlations between variables were examined with Pearson’s correlation coefficient. Statistical significance was assumed when the null hypothesis could be rejected at p < 0.05. Statistical analyses were conducted with SPSS Statistics version 26.0 (IBM, Armonk, NY, USA) and GraphPad Prism 9.0.0.

## Results

**Fig. 1:**
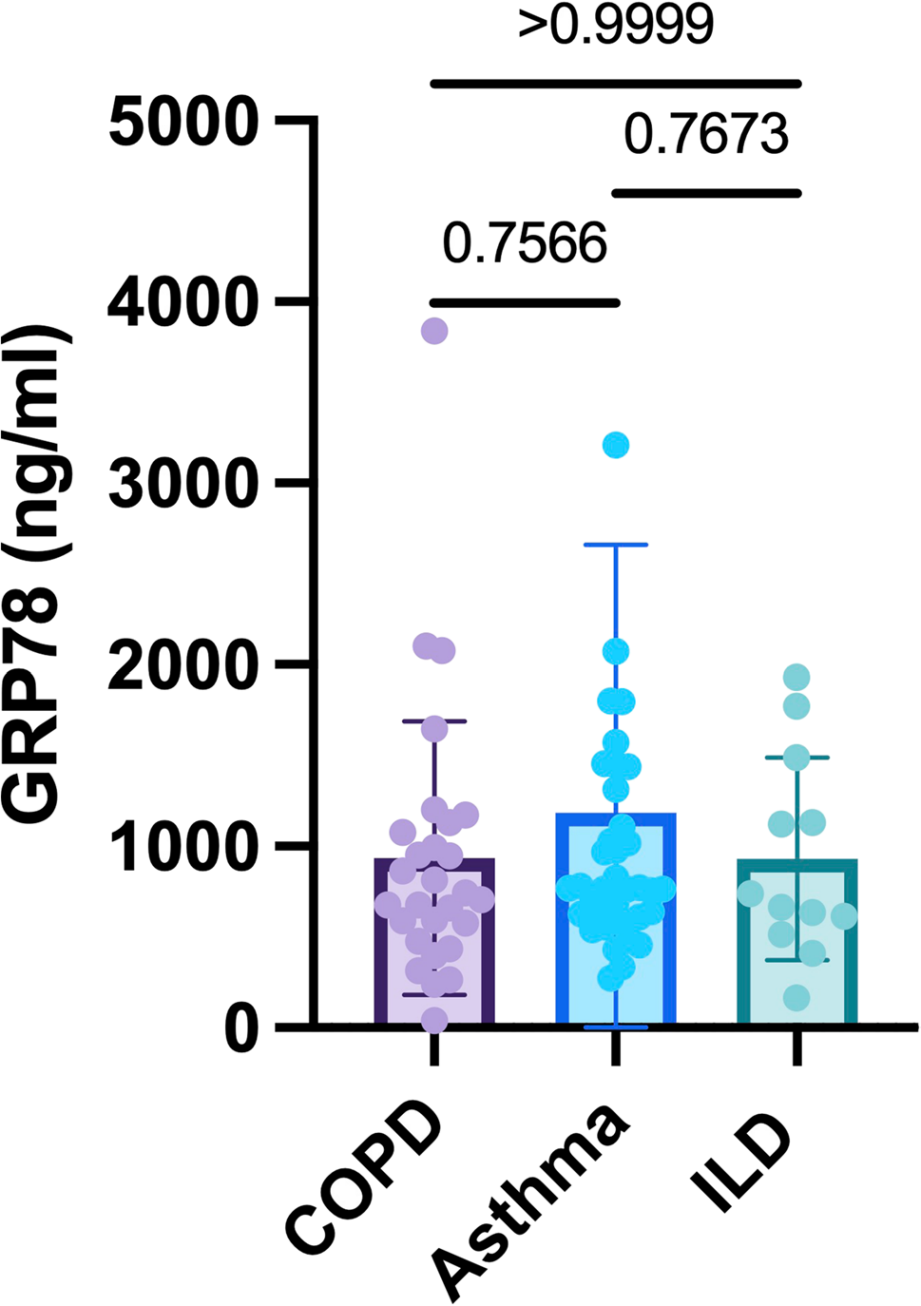
GRP78 serum levels as a function of the underlying pulmonary disease entity. Abbreviations: COPD: chronic obstructive pulmonary disease; ILD: interstitial lung disease

Table 1 summarizes the demographic and clinical characteristics of study participants, stratified by biomarker results. Overall, patients were middle-aged (59.8 ± 12.4 years), the gender ratio was largely balanced (48.7% female). 28 out of 78 patients (35.9%) suffered from COPD, asthma was present in 38/78 patients (48.7%); the remaining 12/78 patients (15.4%) offered ILD. Intercohortal comparison as a function of the underlying pulmonary disease entity evidenced no substantial differences in GRP concentrations (Figure 1). The entire study population was stratified into two groups according to the median GRP78 plasma level of 763.6 ng/ml. Comparison of clinical data in the resultant two patient groups revealed a significantly higher capillary pO_2_ amongst patients with elevated GRP78 levels (75.3 ± 11.7 mmHg vs. 67.8 ± 15.9 mmHg, *p* = 0.02). In Table 2, pulmonary function parameters are summarized. Patients with elevated GRP78 levels had a significantly higher total lung capacity (% of predicted TLC; 114.08 ± 18.9 % vs. 103.43 ± 23.4 %, *p* = 0.04) and a higher residual volume (4.11 ± 1.3 L vs. 3.40 ± 1.5 L, *p* = 0.03). However, there was no significant difference when the residual volume was analysed as a percentage of predicted volume.

**Table 1:** Demographic and clinical data of study population, stratified by GRP78 measurements

	All patients (n = 78)	GRP78 < median (n = 39)	GRP78 > median (n = 39)	*p*-value*
Demographics				
Age [years]	59.8 ± 12.4	58.23 ± 12.6	61.4 ± 12.1	0.27
Female	38 (48.7%)	18 (46.2 %)	20 (51.3 %)	0.82^F^
Smoking habits				
Never	34 (43.6%)	15 (38.5 %)	19 (48.7 %)	0.49^F^
Former smoker	32 (41.0%)	16 (41.0 %)	16 (41.0 %)	
Current smoker	12 (15.4%)	8 (20.5 %)	4 (10.3 %)	
Packyears	6.5 (0–40)	15 (0–40)	2 (0–40)	0.31^M^
Capillary blood gas analysis				
Capillary pH	7.44 ± 0.35	7.44 ± 0.03	7.45 ± 0.04	**0.04**
Capillary pO_2_ [mmHg]	71.6 ± 14.4	67.8 ± 15.9	75.3 ± 11.7	**0.02**
Capillary pCO_2_ [mmHg]	34.8 ± 4.3	35.5 ± 4.1	34.1 ± 4.3	0.17
Frequent exacerbator	44 (56.4%)	18 (46.2 %)	26 (66.7 %)	0.11^F^

Data are presented as n (%), mean ± standard deviation or median (interquartile range, quartile 1/quartile 3). *P-value refers to data comparison between patients, stratified by the median GRP78 plasma level of 763.6 ng/ml (GRP78 < 763.6 ng/ml vs. GRP78 ≥763.6 ng/ml). Unpaired *t*-test was applied, if not otherwise stated (^F^ = Fisher´s exact test, ^M^ = Mann–Whitney *U*-test). Statistically significant differences are given in bold.

Abbreviations: *DL*_*CO*_, diffusion capacity of the lung for carbon monoxide; *FEV1*, forced expiratory volume in 1 s; *pCO*_*2*_, carbon dioxide tension; *pO*_*2*_, oxygen tension

**Table 2:** Pulmonary function parameters of study population, stratified by GRP78 measurements

	All patients (n = 78)	GRP78 < median (n = 39)	GRP78 > median (n = 39)	*p*-value*
FEV_1_ [L]	1.81 ± 0.97	1.81 ± 0.87	1.80 ± 1.0	0.97
FEV_1_ [% predicted]	59.4 ± 25.4	56.46 ± 22.1	62.43 ± 28.2	0.30
DL_CO_ [% predicted]	52.6 ± 25.4	54.06 ± 23.4	51.14 ± 27.5	0.63
TLC [L]	6.82 ± 1.7	6.34 ± 1.6	7.02 ± 1.7	0.08
TLC [% predicted]	109.9 ± 21.8	103.43 ± 23.4	114.08 ± 18.9	**0.04**
VC [L]	2.95 ± 1.04	2.94 ± 1.1	2.91 ± 1.1	0.89
VC [% predicted]	76.73 ± 21.9	76.35 ± 24.5	76.58 ± 20.3	0.97
FVC [L]	2.80 ± 1.0	2.81 ± 1.0	2.73 ± 1.1	0.33
FVC [% predicted]	69.0 ± 20.4	68.94 ± 21.6	67.97 ± 19.9	0.84
RV [L]	3.87 ± 1.5	3.40 ± 1.5	4.11 ± 1.3	**0.03**
RV [% predicted]	175.1 ± 61.7	159.27 ± 68.6	184.44 ± 50.2	0.08

Data are presented as n (%), mean ± standard *P-value refers to data comparison between patients, stratified by the median GRP78 plasma level of 763.6 ng/ml (GRP78 < 763.6 ng/ml vs. GRP78 ≥763.6 ng/ml). Unpaired *t*-test was applied.

Abbreviations: *DL*_*CO*_, diffusion capacity of the lung for carbon monoxide; *FEV1*, forced expiratory volume in 1 s; *TLC*, total lung capacity; *VC*, vital capacity; *FVC*, forced vital capacity; *RV*, residual volume

**Fig. 2:**
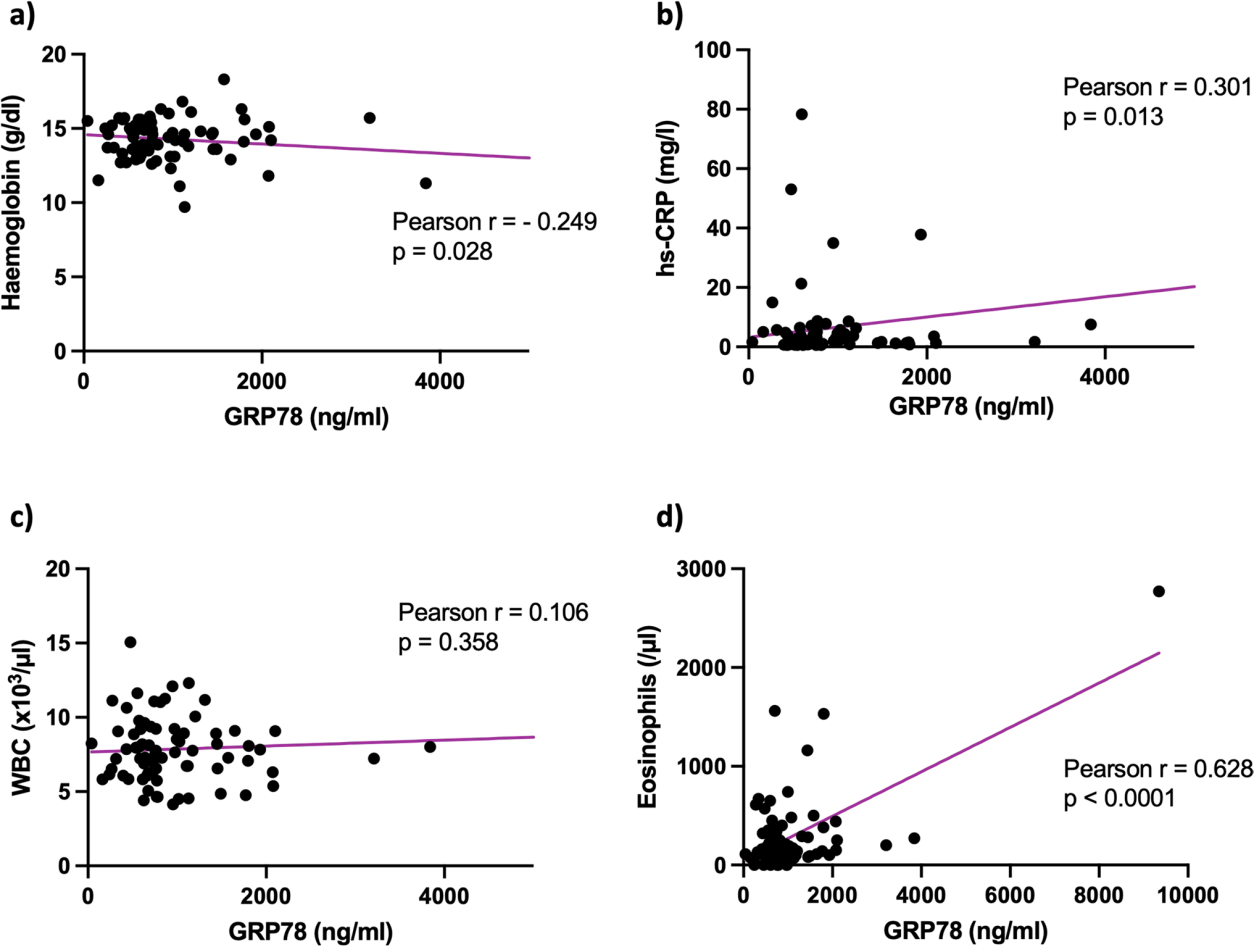
Correlation analysis between GRP78 serum concentration and (**a**) haemoglobin, (**b**) high-sensitivity C-reactive protein, (**c**) white blood cell count and (**d**) eosinophils. Abbreviations: hs-CRP: high-sensitivity C-reactive protein; WBC: white blood cell count

Statistically significant correlations were observed between GRP78 concentrations, on the one hand, and haemoglobin, hs-CRP and eosinophil counts, on the other hand (haemoglobin: Pearson’s *r* = −0.25, *p* = 0.03; hs-CRP: Pearson’s *r* = 0.30, *p* = 0.01; eosinophils: Pearson’s *r* = 0.63, *p* < 0.0001; Figure 2). By contrast, total leukocyte count was not related to GRP78 plasma level (Pearson’s *r* = 0.11, *p* = 0.36).

**Fig. 3:**
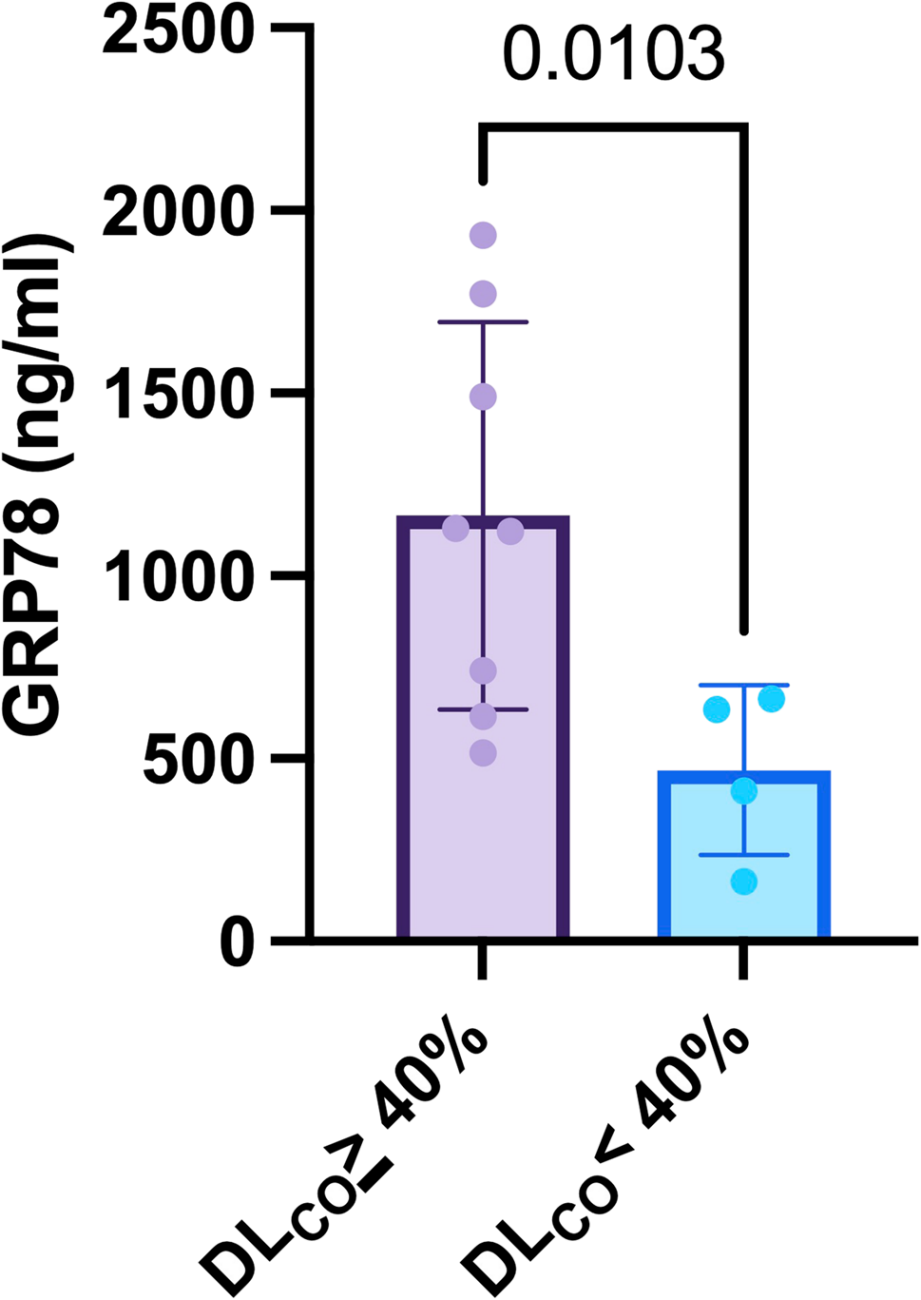
GRP78 serum levels in ILD patients with severe reduction in diffusion capacity (DLCO < 40% of predicted). Abbreviations: DLCO: diffusion capacity for carbon monoxide; ILD: interstitial lung disease

Subsequently, we evaluated GRP78 measurements in function of severity stratifiers of the specific underlying pulmonary disease. In terms of diffusion capacity, a DL_CO_ below 40% of the predicted value discriminates between severe and moderate diffusion reduction and is an established definer of advanced ILD. In our ILD cohort, patients with a DL_CO_ <40% of predicted exhibited a significant decrease in GRP78 levels (524 ng/ml (226-656) vs. 1126 ng/ml (647–1701), *p* = 0.01; Figure 3). In ILD patients, GRP78 levels positively correlated with the DL_CO_ (Pearson’s *r* = 0.58, *p* = 0.048).

**Fig. 4:**
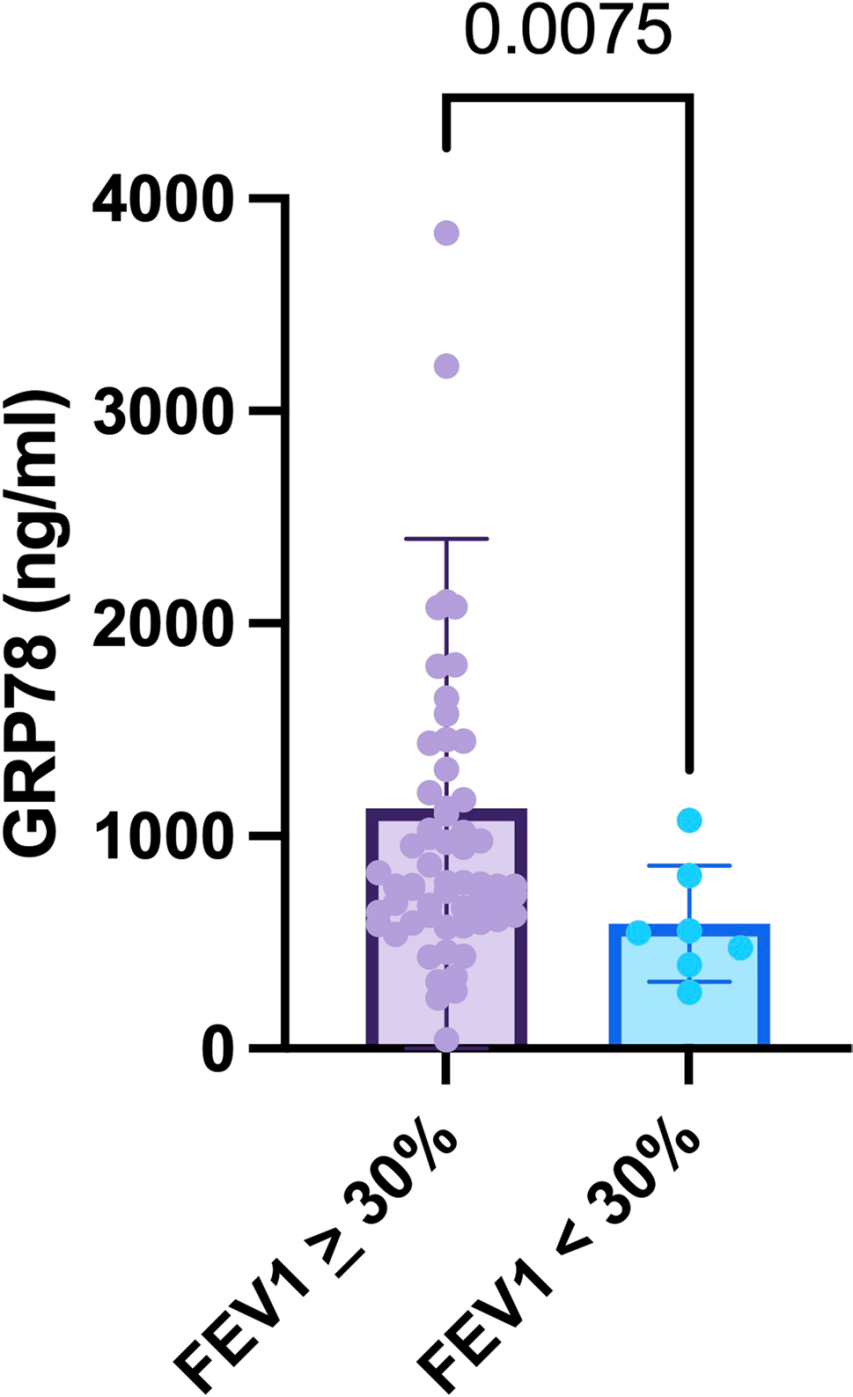
GRP78 serum levels in COPD and asthma patients with severe obstructive airflow limitation (FEV1 <30% of the predicted value). Abbreviations: COPD: chronic obstructive pulmonary diseas; FEV1 : forced expiratory volume in one second

In COPD and asthma, both disease entities characterized by obstructive ventilatory defects, a FEV_1_ <30% of the predicted value marks severe obstructive airflow limitation (Global Strategy for Prevention, 2023; Global Initiative for Asthma: 2022 Main Report, 2023). When transferred to our COPD and asthma study population, significantly lower GRP78 levels were measured in patients with a FEV_1_ <30% of predicted (*p* = 0.0075, Figure 4).

**Fig. 5:**
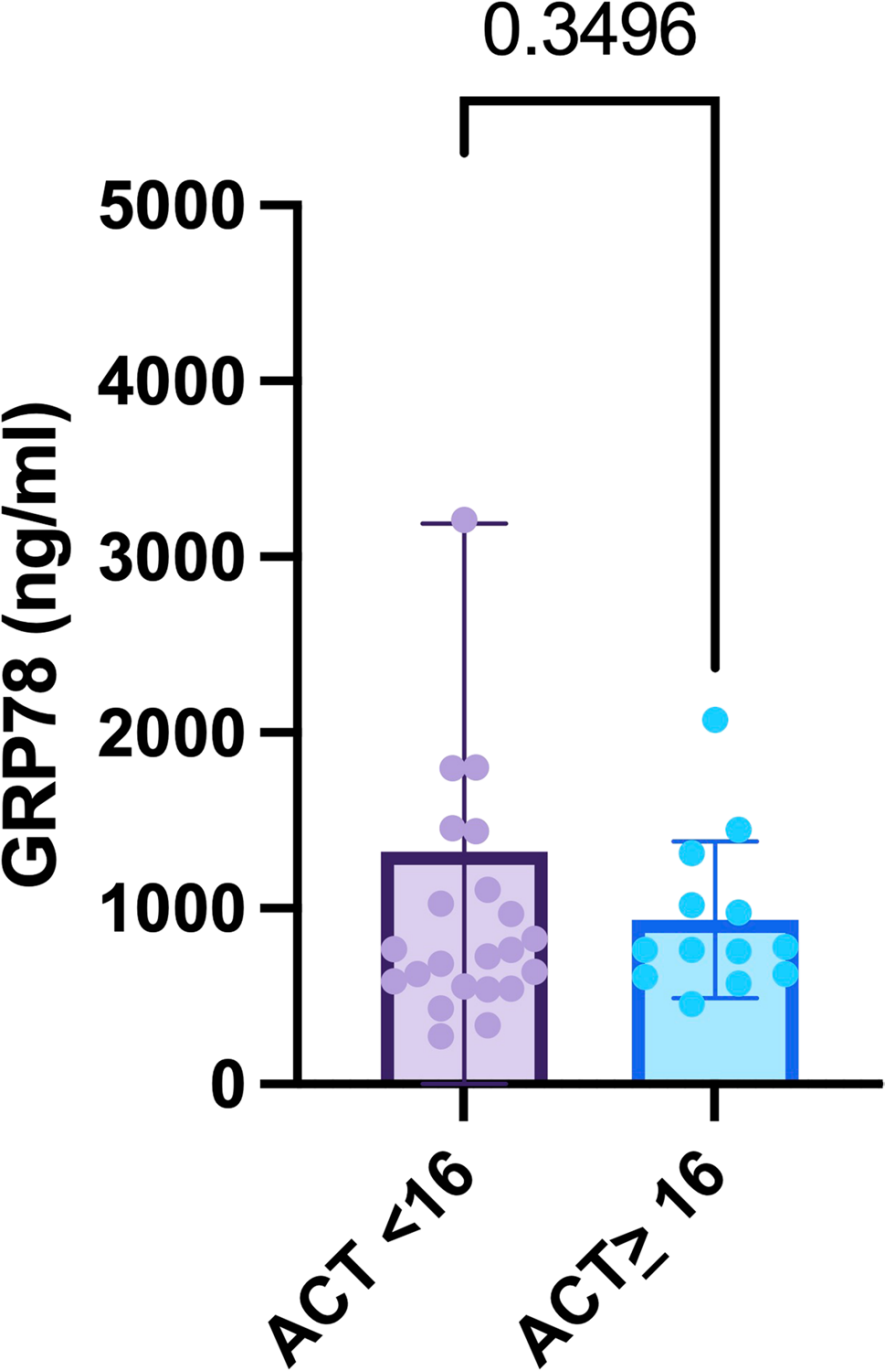
GRP78 serum levels in asthma patients lacking disease control (ACT < 16). Abbreviations: ACT: asthma control test

With a view to symptom burden, the COPD Assessment Test (CAT) and the Asthma Control Test (ACT) were performed, both validated questionnaires to evaluate symptomatology in COPD and asthma, respectively. A CAT-score ≥10 and an ACT-score <16 indicate lacking disease control. As to our asthma cohort, symptom burden showed no association with GRP78 measurements (*p* = 0.35, Figure 5). Regarding our COPD cohort, all patients included into the study stated poor disease control (CAT-score ≥10), irrespective of GRP78-levels.

## Discussion

In the present study, we aimed to determine the potential of serum GRP78 as a biomarker in chronic pulmonary diseases encompassing COPD, asthma and ILD. The main findings of our study are as follows: (i) Increased GRP78 concentrations were accompanied by improved oxygenation status. (ii) In terms of severity of the specific underlying disease entity, decreased GRP78 levels were associated with severity of obstructive airflow limitation in COPD and asthma, on the one hand, and with severity of diffusion capacity limitation in ILD, on the other hand. In light of this association between elevated GRP78 protein expression and a favourable disease course, a protective effect of GRP78 on pulmonary disorders is presumable.

Given its vast surface area of approximately 100 m^2^ and the high volume of inhaled air, the lung is continuously exposed to noxae including environmental pollutants, cigarette smoke, pathogens and allergens. Accumulating evidence suggests that these constant environmental threats may elicit protein misfolding and subsequently activate the UPR signaling pathway (Osorio et al., 2013). COPD constitutes a chronic, debilitating pulmonary disorder of multicomponent nature, defined by chronic airway inflammation (Aksoy et al., 2017). This inflammatory response, resultant from exposure to noxious fumes and airborne particles, is noticeable at both a local and systemic level (Oudijk et al., 2003). Recently, considerable efforts have been made to clarify the role of the ER stress-UPR axis in COPD (Barreiro et al., 2019; Naiel et al., 2020). Airway cell damage in COPD is mainly attributable to oxidative stress, originated by inhaled environmental triggers. Oxidants (e.g., reactive oxygen species) mediate oxidative stress response, leading to activation of the UPR pathway (Aghaei et al., 2020). However, the precise downstream regulatory interactions that allow for recognition and handling of these inhaled noxae remain undefined. Enhanced concentrations of GRP78, a major player in the UPR, has been ascertained in the bronchoalveolar lavage fluid and lung specimens of active smokers (Aksoy et al., 2017; Kelsen et al., 2008). In COPD, GRP78 has been reported as a potential biomarker, correlating with both FEV_1_ and the extent of emphysema (Merali et al., 2014). Similarly, we presently observed altered GRP78 concentrations dependent on the severity of the underlying obstructive pulmonary disorder. In case of severe airflow obstruction, defined by a FEV_1_ <30% of the predicted value, GRP78 was significantly reduced. This observation is in line with the assumption that increased GRP78 expression mitigates ER stress-induced lung inflammation and apoptosis and thereby exhibits protective effects in inflammatory pulmonary diseases.

Beyond COPD, asthma represents the second most frequent obstructive respiratory disorder, likewise characterized by chronic airway inflammation. As a function of the underlying disease processes that determine clinical and pathophysiological features, a distinction is made between diverse asthma phenotypes (*Global Initiative for Asthma: 2022 Main Report*, 2023). They encompass the broad range of allergic, eosinophilic and neutrophilic asthma. Allergic asthma is defined by the induction of IgE antibodies subsequent to allergen exposition. Most elicitors of allergic asthma are capable of inducing ER stress-UPR response. Pathinayake and colleagues demonstrated evidence of ER stress/UPR in induced sputum and bronchoscopy specimens of patients with asthma (Pathinayake et al., 2022). ER stress was mainly present in those patients with active eosinophilic and neutrophilic airway inflammation. Moreover, GRP78 gene expression was associated with lung function decline. Consistently, we measured lowest GRP78 levels in patients with severe airway obstruction, as assessed by pulmonary function testing. Remarkably, the positive correlation observed between GRP78 and eosinophil counts in the entire patient population was solely driven by the asthma cohort (Pearson’s *r* = 0.79, *p* < 0.001). To address the role of eosinophils in allergic inflammation, Wang et al. challenged eosinophil-deficient mice with *Aspergillus fumigatus*, resulting in fungus-induced allergic lung inflammation (Wang et al., 2021). They ascertained decreased GRP78 protein levels in bronchial epithelial cells of fungus-exposed, eosinophil-deficient mice as compared to fungus-exposed, eosinophil-sufficient wild-type mice. Additionally, at a lung gene level, GRP78 was upregulated in challenged wild-type mice. Increased apoptosis was found in both challenged eosinophil-deficient and -sufficient mice, though the latter displayed a milder apoptotic cell activity. These findings support the notion that eosinophils promote an upregulation of the UPR pathway and are detrimental in allergic pulmonary response. Notwithstanding, the question whether UPR activation negatively impacts cellular survival or rather intends restoration of cellular damage control in eosinophilic allergic lung inflammation remains to be answered.

ILD encompasses a large group of more than 200 parenchymal lung diseases that share similar clinical, radiological and pathophysiological characteristics (Travis et al., 2013). A subset of ILD patients may develop progressive pulmonary fibrosis. Idiopathic pulmonary fibrosis (IPF) represents the archetypal progressive fibrotic ILD characterized by relentless lung function decline and earlier mortality (Raghu et al., 2022). In terms of ER stress and UPR activation in ILD, most evidence exists for IPF. Increased ER stress-UPR response including enhanced GRP78 expression has been found in the alveolar epithelium of IPF patients (Lawson et al., 2008). However, UPR signature is not limited to the alveolar epithelium, but also prominent in subjacent fibroblast foci, supporting a correlation between ER stress and IPF pathogenesis. Exogenous stimulants known to increase the probability of developing IPF in genetically susceptible individuals, comprise cigarette smoke, viral infections, microaspiration, airborne pollutants and occupational exposures (Wolters et al., 2018). Noteworthy, many of these risk factors are established ER stressors, indicating that ER stress and UPR appear to be critically involved in IPF onset and progression. Within our ILD study population, only two patients presented IPF. The remaining ILD study cohort was heterogenous with regard to the underlying ILD aetiology but shared the similarity of being mostly progressive fibrosing in nature. In ILD patients, GRP78 levels positively correlated with DL_CO_ (Pearson’s *r* = 0.58, *p* = 0.048). This association was particularly noticeable in those patients with severe diffusion capacity impairment, defined by a DL_CO_ <40% of the predicted value. To the best of our knowledge, no data exist by now evaluating the relation between pulmonary function decline in ILD and chaperone levels. Diffusion capacity represents an established physiological marker of disease severity and progression in ILD. Our finding of a positive correlation between GRP78 and diffusion capacity is consistent with our aforementioned results in obstructive ventilatory defects, in which disease severity is mainly defined by the degree of obstructive airflow limitation, quantified by FEV_1_. In both obstructive and restrictive pulmonary disorders, GRP78 protein concentrations were reduced with increasing disease severity. In keeping with this, we ascertained the highest GRP78 levels in patients with best oxygenation status. While clearly, we cannot address the mechanisms underlying this suggestion, these data enhance the idea that GRP78 may exert protective effects on the course of chronic inflammatory pulmonary diseases like COPD, asthma or ILD.

Our study has several limitations including its single-centre design that impeded the inclusion of a larger number of patients. The study population was composed of patients exhibiting three distinct chronic pulmonary illnesses. Moreover, within the ILD patient cohort, heterogeneity arose from the varying ILD-underlying aetiologies. However, these aetiological variations were potentially overcome by the commonality of a progressive fibrosing conduct. Finally, we confined GRP78 association analyses to pulmonary function and biochemical parameters. Additional chest imaging might have been a valuable adjunct to determine the radiological extent of disease severity.

Our results suggest that GRP78 could be clinically utilized as an additional marker for performing risk stratification in patients with pulmonary diseases. Patients with low GRP78 levels might benefit from closer supervision to detect clinical deterioration. Additionally, we plan to conduct larger follow-up studies to examine prognostic properties of GRP78 in patients with pulmonary disorders. These results will provide further evidence to the significance of GRP78 as biomarker in patients with lung diseases.

In conclusion, the present study implicates GRP78, a hallmark effector of the response to ER stress, in the progression of chronic pulmonary diseases. Elevated GRP78 levels were associated with a more favourable disease course. Taken together, these data support the concept of a presumably protective role of GRP78 in the presently studied pulmonary disorders. Simultaneously, they set the stage for larger studies including disease follow-up.
